# Selective laser trabeculoplasty success in pediatric patients with glaucoma: two case reports

**DOI:** 10.1186/1752-1947-7-198

**Published:** 2013-07-26

**Authors:** Julia Song, Alice Song, Trisa Palmares, Michael Song

**Affiliations:** 1 Long Beach Memorial Medical Center, 2840 Long Beach Boulevard, #330, Long Beach, CA 90806, USA

## Abstract

**Introduction:**

Selective laser trabeculoplasty is a treatment option to lower intraocular pressure in patients with glaucoma. It has been proven to work in adults. We describe two pediatric patients with glaucoma who responded well to selective laser trabeculoplasty.

**Case presentations:**

Two patients with pediatric glaucoma underwent selective laser trabeculoplasty. Patient 1 was a nine-year-old Iranian Asian girl with secondary aphakic glaucoma who was taking four glaucoma medications. She had a 50% decrease in intraocular pressure five weeks after selective laser trabeculoplasty. She was able to discontinue all four glaucoma drops after treatment. Patient 2 was a 13-year-old Filipino Caucasian boy who presented with early juvenile open-angle glaucoma and who was on no medications. He had a 40% drop in intraocular pressure four weeks after selective laser trabeculoplasty.

**Conclusion:**

Selective laser trabeculoplasty can decrease intraocular pressure in pediatric patients with glaucoma, both as a primary and secondary therapy. This study demonstrates that selective laser trabeculoplasty is a good option for the treatment of glaucoma in the pediatric population.

## Introduction

Selective laser trabeculoplasty (SLT) was developed in 1999 to reduce intraocular pressure (IOP) in patients with glaucoma [1]. Because SLT selectively targets the pigmented trabecular meshwork cells, it is a more gentle treatment than argon laser trabeculoplasty. There is no histologic scarring or coagulative damage to the trabecular meshwork [2], thus reducing collateral damage to surrounding tissues. The incidence of iritis and elevated IOP is much lower compared with argon laser trabeculoplasty [3]. In adults, SLT has a variable success rate (40% to 70%) [4-6] but has been shown to be safe [6]. SLT is also safe as initial therapy in both open-angle glaucoma and patients with ocular hypertension [7,8].

The incidence of congenital cataracts is 1.2 to 6 cases per 10,000. Congenital glaucoma is rare. Primary congenital glaucoma is rare, occurring in one in 10,000 births. Secondary congenital glaucoma can follow cataract extraction in infants. Congenital glaucoma is usually treated surgically, not medically. Laser trabeculoplasty is typically not successful in aphakic or congenital glaucoma [9].

## Case presentations

### Patient 1

The patient was a nine-year-old Iranian Asian girl with aphakic congenital glaucoma OS. Her past medical history was non-contributory. Her past ocular history was significant for congenital cataracts; she was s/p cataract extraction in infancy in both eyes. Her family history was significant for a younger brother with congenital cataracts who also underwent cataract extraction in both eyes but did not develop glaucoma.

Our patient had been referred for elevated IOP in her left eye. Her visual acuity was 20/20 in each eye. Preoperatively, her average IOPs were 15mmHg in her right eye and 23mmHg in her left eye, recorded by the same examiner. She was on four topical glaucoma medications for her left eye: timolol-brimonidine (Combigan®, Allergan, Irvine, CA, USA) three times a day, brinzolamide (Azopt®, Alcon, Fort Worth, TX, USA) three times a day and bimatoprost (Lumigan®, Allergan) every bedtime. Slit lamp examination revealed aphakia in both eyes. Gonioscopy revealed open anterior chamber angles to grade IV with rare pigment in both eyes and rare peripheral anterior synechiae in her left eye. Dilated fundus examination showed optic nerve cupping of 0.2 in both eyes, with normal vessels, maculae and periphery. Corneal thicknesses were 546μm and 588μm. Visual field testing showed a full visual field in her right eye and an early inferior arcuate defect in her left eye. Heidelberg retinal tomography showed a full nerve fiber layer in her right eye but a very thin nerve fiber layer in her left eye.

After informed consent was obtained from her mother, our patient underwent SLT in her left eye with settings of: 0.5mJ to 0.9mJ, 80 spots, 360°, 0.3 nanoseconds. She tolerated the procedure well without side effects.

By postoperative week 5, our patient had a 50% drop in her IOP from 23mmHg to 9mmHg. Over the next few months, she was able to discontinue all four of her topical glaucoma medications. Her response to SLT was sustained for 10 months. She subsequently underwent a second SLT treatment in her left eye with a modest IOP drop. Her IOP remained stable six months after the second SLT treatment (14 months after the first SLT treatment), and the visual field defect in her left eye improved and normalized one year later. Eight months after the second SLT treatment in her left eye, her IOPs were stable at 18mmHg in both eyes.

### Patient 2

The second patient was a 13-year-old Filipino Caucasian boy with open-angle glaucoma (more in his right eye than left). His past medical history was noncontributory. His past ocular history was negative for trauma or surgery. His family history was negative for glaucoma.

His visual acuity was 20/20 in each eye. Preoperatively, his IOPs were on average 23mmHg in his right eye and 19mmHg in his left eye, recorded by the same examiner. He was on no glaucoma medications. Slit lamp examination revealed deep and quiet anterior chambers and clear lenses in both eyes. Gonioscopy revealed open anterior chamber angles to grade IV OU with rare pigment. A dilated fundus examination revealed optic nerve cupping of 0.9 in both eyes, with normal vessels, maculae and periphery. Ancillary testing revealed normal corneal thicknesses of 562μm and 555μm. Visual field testing revealed a superonasal step and an enlarged blind spot in his right eye and full visual field in his left eye. Heidelberg retinal tomography revealed very thin nerve layers superotemporally and inferotemporally in his right eye and a very thin nerve layer superotemporally in his left eye.

Our patient did not want to start topical glaucoma medications. After informed consent was obtained from his mother, our patient underwent selective SLT in his right eye with settings of: 0.8mJ, 100 spots, 360°, 0.3 nanoseconds. He tolerated the procedure well without side effects.

By postoperative week 4, our patient had a 39% drop in his IOP from 23mmHg to 14mmHg. His IOPs were 17mmHg and 21mmHg, respectively at four months; by six months, the IOP was 19mmHg in both eyes (still lower than the baseline of 23mmHg in his right eye).

## Discussion

These are the first reported two cases of SLT success in pediatric patients with glaucoma (one with secondary aphakic glaucoma and one with juvenile open-angle glaucoma). Both patients had significant reductions in IOP after SLT (50% and 39%). Our first patient was able to discontinue all four topical medications. Our second patient did not need to use topical glaucoma medication.

This study demonstrates that SLT may be a viable treatment option prior to incisional glaucoma treatment or glaucoma medications. It also demonstrates that SLT is efficacious as an adjunct or as primary therapy [10]. As in adults, SLT in the pediatric population can be used to help patients discontinue glaucoma medications [11]. Also as in adults, the efficacy of SLT diminishes over time [12,13]. Our first patient's scenario demonstrated that SLT can be safely repeated, as demonstrated in other studies [14]. Both patients had IOP lowering in their contralateral eyes as well, as demonstrated in prior studies [15]. The limitations of this study include its retrospective nature as well as the small sample size. Our second patient needs longer follow-up. Larger studies should be done to confirm these encouraging results.

## Conclusions

SLT is a safe and efficacious treatment for pediatric patients with glaucoma, both as primary and adjunct therapy.

## Consent

Written informed consent was obtained from both patients' legal guardians for publication of this case report and any accompanying images. A copy of the written consent is available for review by the Editor-in-Chief of this journal.

## Competing interests

The authors declare that they have no competing interests.

## Authors’ contributions

JS examined the patients before and after treatment. MS, AS and TP performed the literature search. All authors read and approved the final manuscript.
